# A Bifunctional Protease Cleaves the Leader Peptide in the Biosynthesis of Class I Microviridins

**DOI:** 10.1002/cbic.70297

**Published:** 2026-03-31

**Authors:** Nico Brüssow, Stella Scholz, Antje Stindt, Vincent Wiebach, Tino Damaszek, Roderich D. Süssmuth, Elke Dittmann, Martin Baunach

**Affiliations:** ^1^ Institut für Biologie und Biochemie Universität Potsdam Potsdam Germany; ^2^ Institut für Chemie Technische Universität Berlin Berlin Germany; ^3^ Institut für Pharmazeutische Biologie Universität Bonn Bonn Germany

**Keywords:** biosynthesis, enzyme catalysis, leader peptide processing, microviridin, pathway reconstitution, protease, ribosomally synthesized and posttranslationally modified peptide

## Abstract

Microviridins are a prominent family of highly potent serine protease inhibitors, of which individual variants specifically inhibit different types of proteases of pharmacological interest. These natural products of cyanobacterial origin belong to the ribosomally synthesized and posttranslationally modified peptides and feature an unusual cage‐like architecture, which is composed of characteristic lactone and lactam rings. While the modifying enzymes introducing the posttranslational modifications in the course of microviridin biosynthesis are well investigated, the removal of their N‐terminal leader peptides by designated proteases—a key step during maturation—remains enigmatic. In this study, a bioinformatic approach led to the discovery of NosP, which was confirmed as the first specific protease involved in microviridin biosynthesis. In vitro assays with modified precursor peptide, which was obtained from in vitro pathway reconstruction, revealed that NosP is a bifunctional protease, with both endo‐ and aminopeptidase activities. These results, together with the finding that corresponding homologous leader peptides are widespread in cyanobacteria, may pave the way for the efficient production and bioengineering of class I microviridins in vivo and in vitro.

## Graphical abstract

Biochemical characterization of the peptidase candidate NosP reveals bifunctional endo‐ and aminopeptidase activity in the leader peptide removal of class I microviridins from *Nostoc punctiforme*. The discovery of the first leader‐processing peptidase from microviridin biosynthesis may pave the way for the efficient production and bioengineering of class I microviridins in vivo and in vitro.

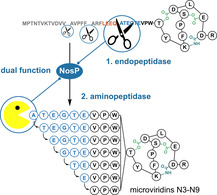

## 1 Introduction

Microviridins, founding member of the graspetide family, are ribosomally synthesized and post‐translationally modified peptides (RiPPs) that were first identified in cyanobacteria [1, 2]. These compounds act as potent and selective inhibitors of various serine proteases, including trypsin, chymotrypsin, subtilisin, and elastase [3]. Owing to their inhibitory activity toward elastase, microviridins have been proposed as potential therapeutic agents for the treatment of pulmonary emphysema [3]. Structurally, microviridins are characterized by a distinctive cage‐like structure comprising two lactone linkages—formed between the side‐chain carboxy group of Asp/Glu and the hydroxy group of Ser/Thr—and a single lactam linkage connecting the *δ*‐carboxyl group of Glu to the *ε*‐amino group of Lys. The two ATP‐grasp ligases MvdD and MvdC consecutively install the lactone and lactam structures on the microviridin precursor peptide (MvdE) [1, 2]. The cyclized precursor peptide is proteolytically processed by a yet‐unidentified endopeptidase. The released cyclized core peptide optionally becomes acetylated at the N‐terminus by an *N*‐acetyltransferase that is encoded within the same biosynthetic gene cluster (BGC) [4].

A recent review on RiPP maturation has highlighted that leader peptide removal represents one of the most mechanistically diverse steps in biosynthesis [5]. Dedicated proteases from various enzyme families including cysteine, serine, and metalloproteases, perform the peptide processing by single‐ or multistep mechanisms that vary in substrate specificity and cellular localization. Notably, many RiPP BGCs, including those of many graspetides, lack an identifiable cognate protease, indicating that leader peptide processing often depends on yet‐uncharacterized enzymes, which often seem not to be encoded in the corresponding BGC [5, 6]. Based on the presence of putative peptidase recognition motifs and number of core peptides, microviridin precursor peptides were subdivided into three classes [7]. Class I precursor peptides consist of a leader peptide that is fused to a single core peptide. This class includes precursors for microviridins A‐K that were originally identified through bioactivity‐guided isolation. Class II precursors may comprise up to five consecutive microviridin‐like core peptides on a single precursor. This group of precursors is exemplified by the precursor peptide AMdnA from *Anabaena* sp. PCC 7120 containing three consecutive microviridin‐like core peptides [8]. Class III precursors contain longer core peptides in which only the C‐terminus clearly resembles a matching microviridin sequence. Representative microviridin‐like peptides were recently characterized for *Chitinophagia japonensis* DSM13484 and designated as chitinoviridines A1 and A2A [9]. While class III precursors are predominantly found in Bacteroidetes, class I and II precursors are particularly common in cyanobacteria. Class II and III precursors comprise double‐glycine motifs and are often accompanied by membrane transport proteins with a cognate C39 peptidase domain. Double‐glycine motifs are also widespread in the larger graspetide family and were detected in about half of the bioinformatically predicted graspetide BGCs. One of these graspetide ABC transporters carrying an N‐terminal C39 domain has recently been characterized biochemically, demonstrating that it cleaves the precursor peptide in vitro after the conserved GG‐motif [10]. Class I microviridin precursors, on the other hand, mostly lack a double‐glycine motif and do not encode potential leader peptidases within their BGCs [7].

Functional expression of the ATP‐grasp ligases MvdC and MvdD in *Escherichia coli* has enabled in vitro reconstitution with a chemically synthesized precursor peptide. The absence of a peptidase within the BGC, however, prevented leader peptide removal [1]. Related studies expressing *mvd* BGCs in *E. coli* encountered similar difficulties: the lack of a cluster‐encoded peptidase led to incomplete maturation and the appearance of multiple side products, suggesting non‐selective processing by endogenous *E. coli* proteases [2, 11]. To overcome this limitation, an in vitro one‐pot system was developed in which the leader peptide was fused directly to the ATP‐grasp ligases, allowing conversion of microviridin core peptides into mature products without the need for a dedicated peptidase [12]. This chemoenzymatic approach was further exploited to introduce functional tags into microviridins, facilitating studies on their still‐elusive ecological roles [13]. Due to the lack of a suitable leader peptidase for class I microviridins, the efficiency of the complementary microviridin in vivo production method falls significantly short of expectations, making it difficult to exploit and screen large libraries of microviridins in hosts such as *E. coli*. This study therefore focused on the bioinformatic search for a suitable leader peptidase for class I microviridins and its subsequent characterization.

## 2 Results and Discussion

### 2.1 Bioinformatic Discovery of the AplP Homolog NosP in Nostoc punctiforme

**FIGURE 1:**
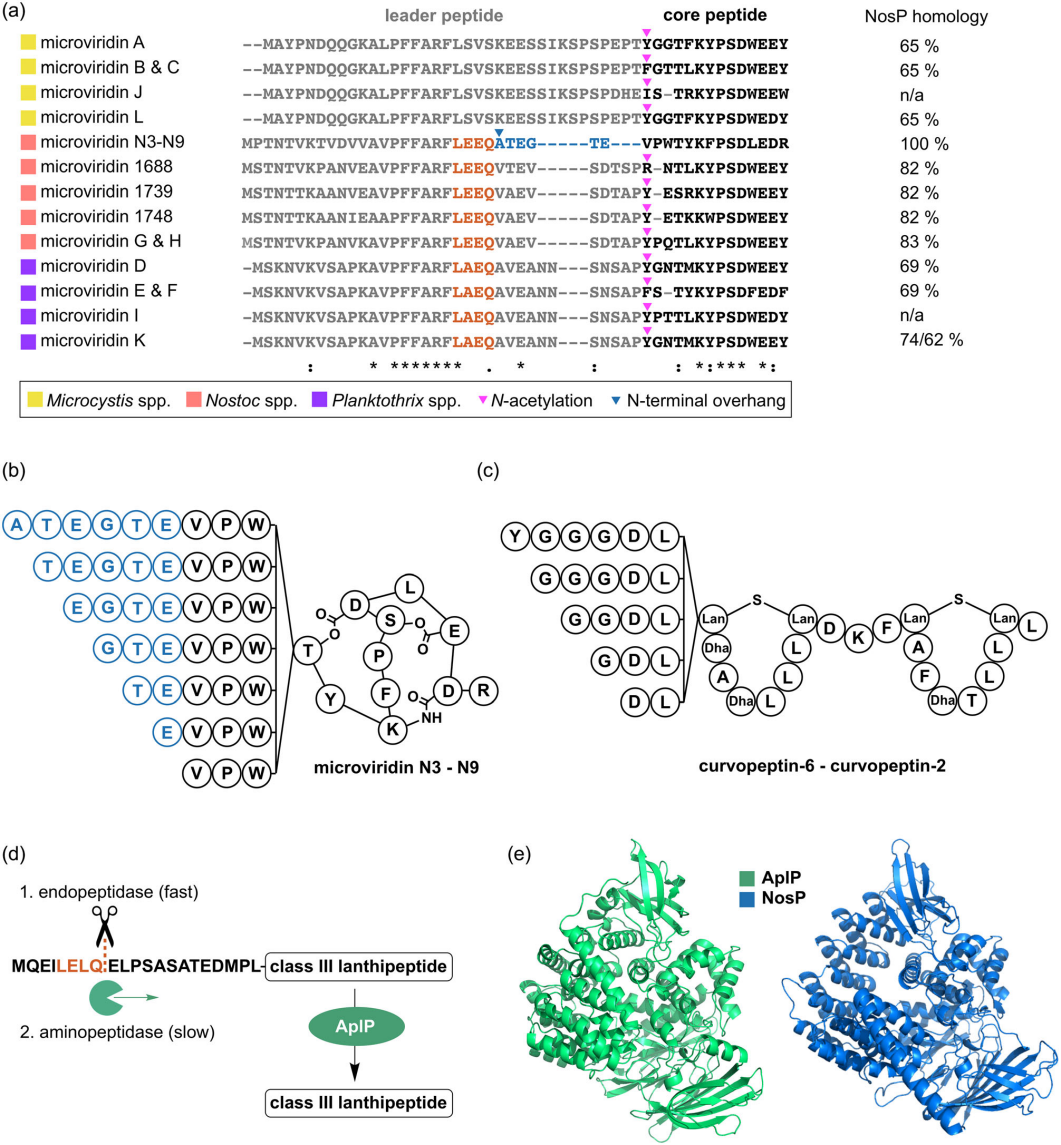
Comparison of microviridin precursor peptides, RiPP structures, and putative leader peptide proteases. (a) Alignment of precursor peptide sequences from all known class I microviridins for which a BGC was identified. The conserved putative cleavage motif (LEEQ/LAEQ) is highlighted in orange. The NosP homolog from the microviridin K producer *Planktothrix agardhii* NIVA‐CYA126/8 is split and encoded in two adjacent ORFs. (b) Structures of microviridins N3‐N9 with the N‐terminal overhang highlighted in blue. (c) Other RiPPs with variable N‐termini as exemplified by the curvopeptins, which are class III lanthipeptides [16]. (d) Leader peptide removal in the biosynthesis of the class III lanthipeptide NAI‐112 by the bifunctional Zn^2+‐^dependent protease AplP with employs endo‐ and aminopeptidase activities [17]. (e) Structural homology between AplP and NosP from *N. punctiforme*. The structures were modeled by *AlphaFold* [18]. Lan – lanthionine; Dha – dehydroalanine. n/a – information not available due to missing genome sequence data.

Analysis of MvdE precursor peptide sequences from all known class I microviridins, for which a BGC can be identified (Figure S1), revealed the existence of two distinct groups of leader peptides. The first group includes known microviridin precursors from *Microcystis* spp. and is characterized by a proline‐rich sequence at the C‐terminus of the leader peptide (Figure 1a). The second group, which is derived from *Nostoc* and *Planktothrix* spp. is clearly distinct and showed congruence only in the conserved PFFARFL motif, which is essential for the activation of the ATP grasp ligases MvdC and MvdD. To date, 17 microviridin congeners have been described from the second group, of which the microviridins produced by *Nostoc punctiforme* PCC 73102 are notable due to a unique structural feature. This strain was reported to generate a mixture of differently processed microviridins, designated N3‐N9, depending on the length of the N‐terminal overhang adjacent to the Thr4‐Asp10 ester (Figure 1b) [14]. This variable N‐terminal length contrasts with other microviridins, which typically possess a clearly defined N‐terminus that is additionally protected by N‐terminal acetylation catalyzed by the GNAT type acetyltransferase MvdB (Figures 1a and S1). Based on the distinct leader peptide characteristics, we postulate that two different types of peptidases mediate the processing of class I microviridins in the different cyanobacterial genera. Notably, this dualism holds true for a multitude of putative novel microviridin precursors, which showcase that a wealth of chemical novelty still awaits discovery (Figure S2).

The simultaneous formation of RiPPs with variable N‐termini has also been described for other RiPP families. In particular, variable length has been observed for class III lanthipeptides, such as labyrinthopeptins and curvopeptins (Figure 1c) [15, 16]. In the case of the class III lanthipeptide NAI‐112, it has been demonstrated that a Zn^2+^‐dependent bifunctional protease, AplP, is responsible for leader peptide processing [17]. AplP was shown to perform partial cleavage of the leader through its endopeptidase activity first, followed by progressive degradation of the remaining leader overhang with its aminopeptidase function (Figure 1d). By analogy, we hypothesized that *N. punctiforme* encodes a similar bifunctional protease with endo‐ and aminopeptidase activities responsible for the stepwise processing of the leader peptide, leading to the formation of multiple microviridin variants (N3‐N9). After re‐inspection of the microviridin leader peptide sequences, we indeed observed a motif similar to the AplP recognition motif upstream of the terminal amino acid of microviridin N9. While AplP cleaves after an LELQ motif, all class I microviridin precursor peptide of the second group possess a highly similar LEEQ/LAEQ motif (Figure 1a). This observation strongly supported the hypothesis that an AplP‐like leader peptidase could be responsible for the processing of the widespread second group of class I microviridins.

A bioinformatic search for an AplP homolog encoded in the genome of *N. punctiforme* PCC 73102 identified the peptidase NosP (*Npun_RS14335*), which is encoded 0.8 Mb distant from the microviridin BGC and annotated as a member of the M1 family metalloproteases. To further validate this finding, bioinformatic analyses were conducted prior to experimental verification of the corresponding peptidase. Comparative genomic analysis revealed that all microviridin producers with an identifiable BGC as well as all investigated strains with putative microviridin BGCs encode NosP homologs (Figures 1a and S2). This consistent cooccurrence suggests a strong genetic association between the microviridin biosynthetic machinery and NosP‐like peptidases encoded in trans. However, several strains harboring NosP homologs lack an identifiable microviridin BGC, and contrary to that, the proline‐rich precursors of the second group might rely on a different kind of protease. This implies that NosP may be involved in the processing of other peptide substrates and represents a more broadly acting peptidase within cyanobacteria. Structural predictions generated using *AlphaFold* [18] revealed a high degree of structural similarity between NosP and AplP, supporting a conserved catalytic architecture (Figure 1e). In addition, sequence alignment of NosP, AplP [17] and aminopeptidase N from *E. coli* [19] confirmed that putative catalytic motifs are conserved in NosP, e.g. the conserved GAMEN motif, which contains a highly conserved Glu residue that is suggested to contribute to the exopeptidase activity of various proteases. This is supposed to occur through interaction of its terminal carboxylic group with the N‐terminus of the substrate [20], or the highly conserved catalytic Zinc‐binding motif HEXXH‐X_18_‐E (Figure S3). Notably, NosP homology correlates with precursor peptide type being highly conserved for class I precursor peptides with an LEEQ motif, moderately conserved for precursor peptides with an LAEQ motif, and less conserved for the second group, which presumably is processed NosP‐independent (Figure 1a). Collectively, these results indicate that NosP is a suitable candidate for processing the *N. punctiforme* microviridin precursor peptide with combined endo‐ and aminopeptidase activity.

### 2.2 Characterization of NosP Endopeptidase Activity

**FIGURE 2:**
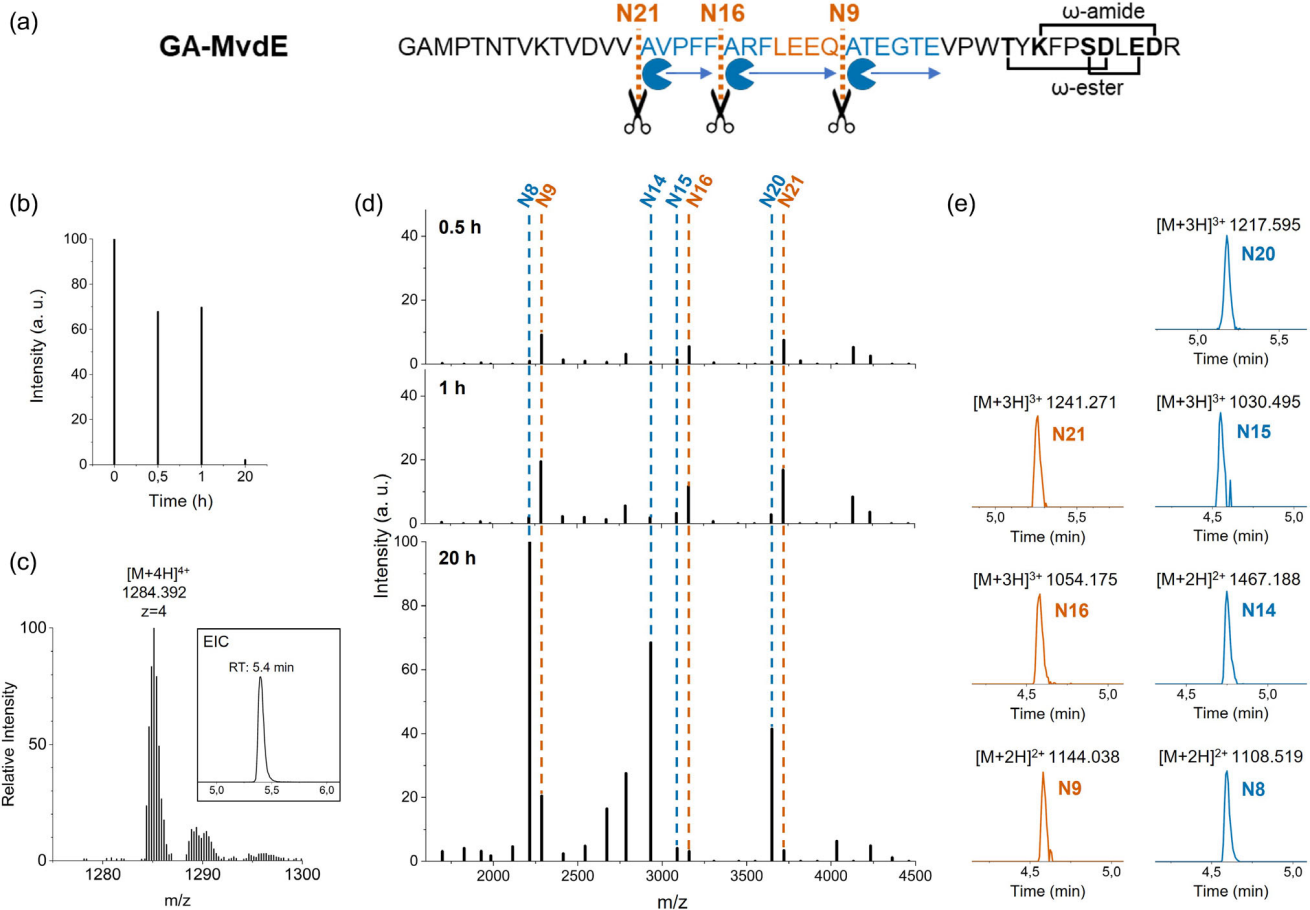
NosP‐mediated cleavage of the microviridin precursor peptide. (a) Sequence of the tricyclic precursor peptide (GA‐MvdE), indicating cleavage sites attributed to the endopeptidase (orange) and aminopeptidase (blue) activities of NosP. (b) Normalized LC‐MS intensities of the precursor peptide showing a time‐dependent decrease during the assay. (c) ESI‐mass spectrum and extracted ion chromatogram (EIC) of the cyclic precursor displaying a peak at *m/z* 1284.392, corresponding to the [M + 4H]^4+^ ion. (d) LC‐MS based quantification of NosP activity on the precursor peptide. Normalized intensities are plotted against *m/z* values for an incubation time of 0.5 h, 1 h and 20 h. The data indicate initial cleavages at three positions, yielding microviridins N9, N16, and N21 consistent with NosP endopeptidase activity (orange). After 20 h, a shift toward the peptides N8, N14, and N20 is observed, consistent with subsequent NosP aminopeptidase activity (blue). (e) EICs of main peptides produced by NosP.

To investigate endopeptidase NosP acting on the microviridin precursor peptide, the full‐length precursor was synthesized using solid‐phase peptide synthesis (Figure S4‐5). Due to solubility problems, the chemically synthesized peptide could not be used. Alternatively, the full‐length precursor was heterologously produced in *E. coli* LOBSTR [21] as a fusion protein with a His_6_‐maltose‐binding protein (MBP), since production of the unfused His_6_‐precursor peptide in *E. coli* did not yield sufficient amounts of soluble precursor peptide (Figure S6). Following expression and purification, MBP was removed by a TEV protease, yielding the full‐length precursor peptide with an additional Gly‐Ala motif at the N‐terminus (Figures 2a and S6b). During RiPP maturation, cleavage of the leader peptide typically represents the final step, occurring only after RiPP‐modifying enzymes have installed the post‐translational modifications on the core peptide. Therefore, in order to prepare a suitable substrate for NosP, the precursor peptide was first enzymatically processed in vitro employing the two ATP‐grasp ligases MvdD and MvdC, originally from *Planktothrix agardhii* NIVA‐CYA 126, that were expressed as His_6_‐fusion proteins (Figure S7). These enzymes have been shown to recognize the highly conserved PFFARFL motif in the leader peptide of microviridins [11]. HPLC‐ESI‐MS analysis of the purified precursor confirmed successful formation of the tricyclic product, revealing a signal at *m/z* 1284.392 corresponding to the expected [M + 4H]^4+^ species (Figure 2c, S8 and S9).

The cyclized precursor peptide was subsequently used as a substrate in in vitro assays with the heterologously produced peptidase NosP (Figures 2 and S6). Normalized LC–MS intensities over time showed a time‐dependent decrease of the precursor peptide in the course of the assay (Figure 2b). LC–MS analysis revealed initial cleavage at three sites within the leader peptide, occurring N‐terminally of position N9, which represents the position adjacent to the conserved LEEQ motif, as well as positions N16 and N21 (Figure 2d). These cleavage products were the predominant species after incubations for 30 min and further increased in abundance after 1 h. Upon extended incubation (20 h), a gradual shift in the cleavage pattern was observed, with the dominant products shifting from N9 to N8, N16 to N14, and N21 to N20. This shift is likely due to the aminopeptidase activity of NosP, resulting in stepwise N‐terminal cleavage of the initial processing products. The observation that NosP cleaves the leader peptide at three distinct sites appears unintuitive. However, a similar pattern has been reported for AplP which cleaves the AplA precursor peptide at four positions [17]. No uniform cleavage motif could be identified for NosP's three endopeptidase cleavage sites, although a general preference for cleavage N‐terminal to Ala residues has been observed for the cyclized MvdE precursor. Furthermore, the reproducible assay results indicate comparatively fast endopeptidase activity of NosP and relatively slow aminopeptidase activity, with only 1–2 amino acids being cleaved off in 24 h. Experiments to test whether NosP requires post‐translational modifications for its activity could not be performed due to the low stability of the unmodified recombinant precursor peptide

### 2.3 Characterization of NosP Aminopeptidase Activity

**FIGURE 3:**
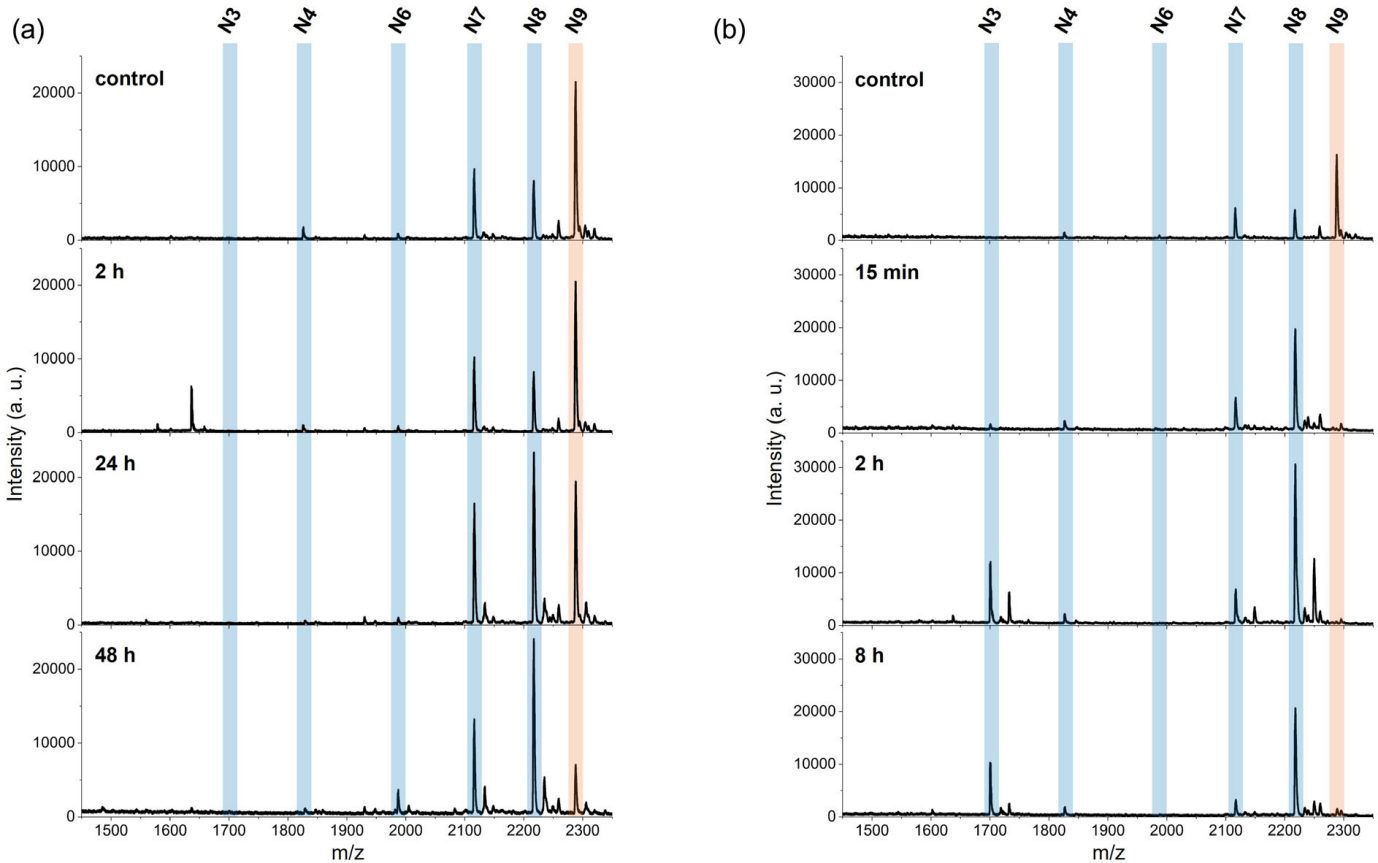
Comparative aminopeptidase activities of NosP and MicP2 on microviridins. (a) MALDI‐TOF mass spectra of the NosP‐mediated digestion of a mixture containing microviridins N7‐N9 purified from *Nostoc punctiforme*. After 24 h, a shift from microviridin N9 to N8 is observed with further conversion toward N6 after 48 h. (b) MALDI‐TOF mass spectra of the MicP2‐mediated digestion. The peptidase MicP2 from *Microcystis arborescens* catalyzes the conversion from N9 to N8 within 15 min and to N3 after 2h, demonstrating that MicP2 acts much faster than NosP.

To further investigate the aminopeptidase activity of NosP, heterologously produced enzyme was incubated with a mixture of microviridins N7‐N9 purified from *N. punctiforme*. As the individual variants coeluted during purification, isolation of pure N9 was not possible. After 24 h of incubation with NosP, MALDI‐TOF‐MS analysis revealed a partial conversion of N9 to N8 by NosP, in agreement with the results observed in the endopeptidase assay, thereby further confirming the aminopeptidase activity of NosP (Figure 3a). Prolonged incubation for 48 h resulted in further processing, with a shift toward the formation of N6. While slow catalytic processing of leader peptides by specific proteases is a common theme in RiPP maturation [5, 22, 23, 24], (e.g., complete removal of the AplA leader peptide by AplP requires extended incubation times of 72 h [17]), NosP processing appeared to be exceptionally slow even by these standards. Thus, we decided to compare NosP with the previously characterized AplP homolog MicP2 from *Microbacterium aborescens*, which, together with its homolog MicP1 is involved in leader peptide removal in the biosynthesis of the strongly antibacterial lanthipeptide microvionin [25]. Both peptidases share a similar cleavage pattern, but MicP2 displays a significantly enhanced processivity and faster turnover than MicP1. In contrast to NosP, MicP2, catalyzed complete conversion of N9 to N8 after only 15 min and further processed microviridins to N3 after only 2 h of incubation (Figure 3b).

Notably, putative cleavage intermediates prior to N3 like N6 and N5 seem to be absent in the MicP2 reaction. This agrees with the previous finding that MicP2 preferentially cleaves C‐terminally of acidic residues, especially Glu, and endopeptidase cleavage after Glu4 for Microviridin N9‐N5 would yield N3. However, the distinctly slower conversion of microviridins by NosP compared to MicP2, (e.g., N9 to N8), indicates that NosP exhibits substantially slower aminopeptidase activity. This slow turnover most likely explains the unprecedented occurrence of multiple microviridin variants (N3‐N9) in *N. punctiforme*, which at first glance appears odd. From a biosynthetic perspective, however, incomplete leader removal might be advantageous for the producer as *N. punctiforme* is the only known microviridin producer that lacks an *N*‐acetyltransferase that normally acetylates the N‐terminus of microviridins yielding acetylated N‐termini of 2–3 amino acids length (Figure S1). While no conclusive SAR studies for microviridins exist that rigorously investigated the impact of these residues on bioactivity, experiments in which N‐terminal Gly of microviridin L was exchanged with Ala indicate an impact of the N‐terminal side chain for the overall bioactivity [26]. In addition, SAR studies with marinostatin – a microviridin‐like serine protease inhibitor that shares microviridins’ lactone ring architecture but lacks the lactam ring – revealed that removal of both N‐terminal amino acids resulted in complete loss of activity [27]. Thus, extended N‐termini as a result of “tamed” endopeptidase activity might compensate for the absence of *N*‐acetylation as a measure to minimize the risk of premature compound degradation by unspecific proteases. This theory is supported by experiments in which the microviridin fraction was incubated with the faster acting endopeptidase homolog MicP2. This partially resulted in the formation of microviridin species with shortened N‐termini (N2‐N1) and even the removal of the complete N‐terminal side chain (N0) upon extended incubation times (Figure S10). Exceptionally slow proteolytic processing of modified precursor peptides, with estimated turnover numbers as low as <10 per day [22] has been observed in a number of other RiPP biosynthetic pathways, e.g. in patellamide biosynthesis and crocagin biosynthesis [5, 22, 23, 24]. This phenomenon has been suggested to be a protective feature of the biosynthetic machinery in preventing premature proteolysis before all required posttranslational modifications have been installed [5, 24]. Self‐protection against self‐inactivation would be a novel complement to the fascinating repertoire of RiPP biosynthesis. However, although fitting well to the natural distribution of the congeners in the native producer, it cannot be ruled out, that the very slow activity is because the protein is not in its native environment and may need protein–protein interactions with other cellular components for more pronounced processivity. In addition, the hypothesis that the unusually slow endopeptidase activity compensates for the absence of a *N*‐acetyltransferase remains experimentally untested at the moment.

**FIGURE 4:**
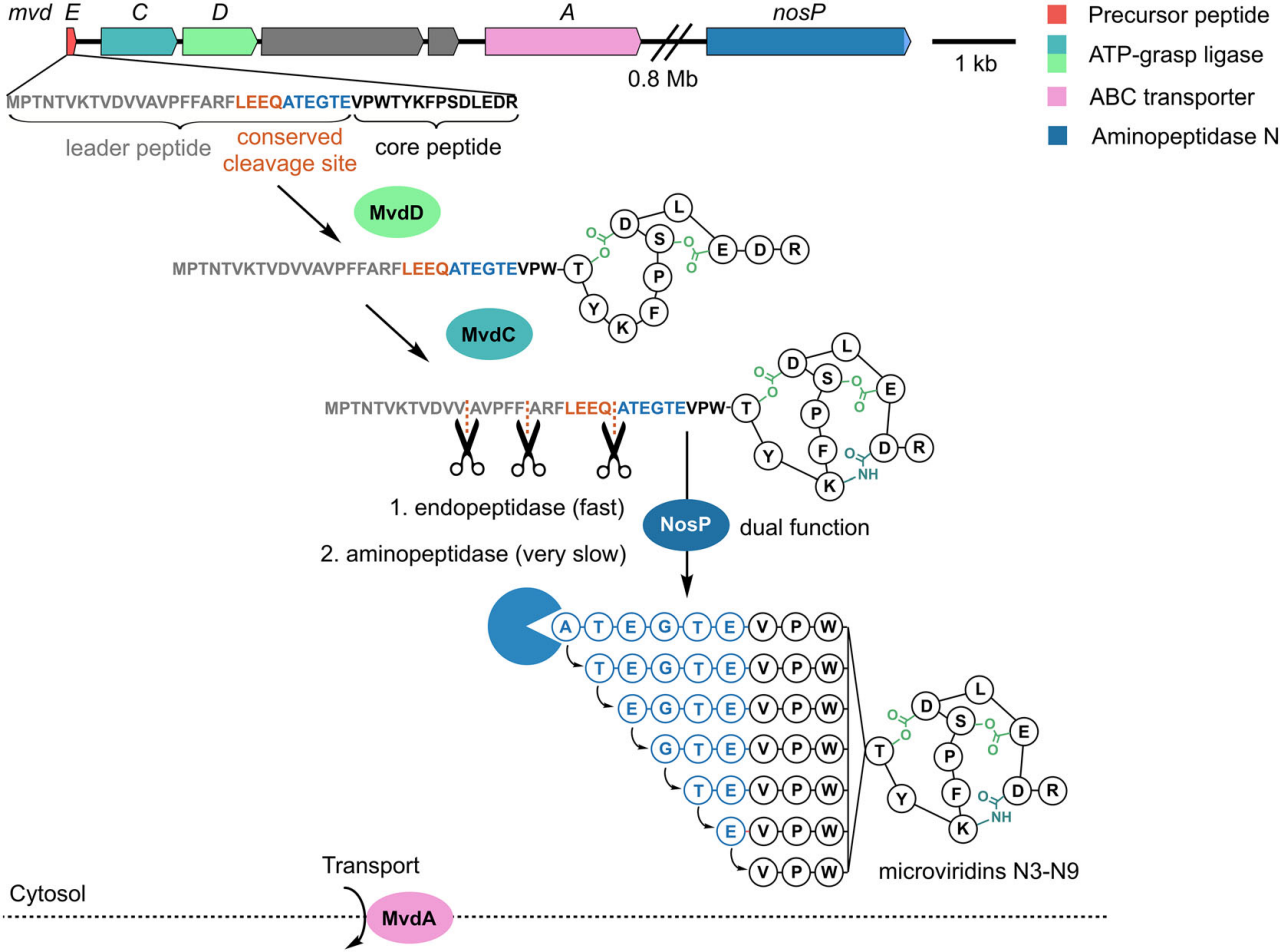
Proposed biosynthesis of microviridins N3‐N9.

Identification and characterization of a specific protease for leader peptide removal in microviridin biosynthesis now allows, for the first time, to postulate a comprehensive biosynthetic pathway. The sequence starts with the formation of the precursor peptide, which is consecutively modified by the two ATP‐grasp ligases MvdD and MvdC to yield the cage‐like core structure by lactone and lactam formation. Finally, the fully maturated precursor peptide is rapidly cleaved by the endopeptidase activity of NosP at three individual sites N‐terminal to Ala residues. This includes proteolytic processing C‐terminally of the conserved cleavage motif LEEQ resulting in the formation of microviridin N9. Subsequently, microviridin N9 is very slowly converted to the microviridin congeners N3‐N8 by the aminopeptidase activity of NosP (Figure 4).

## 3 Conclusion

This study reports the identification of the first specific protease involved in the biosynthesis of microviridins, thereby shedding light on a long‐standing question in the field of RiPP biosynthesis. By employing in vitro pathway reconstruction, modified precursor peptide was obtained, which served as substrate for the in vitro characterization of NosP. Successful bioassays confirmed that NosP has dual functionality, first acting as an endopeptidase that partially removes the leader peptide, followed by an exceptionally slow aminopeptidase activity, yielding microviridin variants with variable N‐termini. The in vitro reconstitution of the entire pathway represents the first reported total biosynthesis of this compound class. Notably, homologous precursor peptides with conserved LEEQ/LAEQ motifs are very widespread in cyanobacteria. Thus, the discovery that two members of the M1 family metalloproteases can process microviridin leader peptides with different endopeptidase preferences and aminopeptidase turnover numbers may pave the way for the efficient production and bioengineering of class I microviridins in vivo and in vitro.

## 4 Material and Methods

### 4.1 Protein Synthesis

Constructs containing genes encoding for the putative peptidase and the precursor peptide were introduced into E. coli LOBSTR. A single colony of transformed E. coli LOBSTR cells was selected from a lysogeny broth (LB) agar plate and inoculated into LB broth supplemented with 40 µL mL−1 kanamycin. The culture was incubated overnight at 37 C and 180 rpm. The overnight culture was then used to inoculate baffled Erlenmeyer flasks containing terrific broth supplemented with the same antibiotic. Expression cultures were grown at 37°C and 200 rpm until reaching an optical density measured at 600 nm (OD600) of 0.8. Protein expression was induced by adding 0.5 mM (final concentration) isopropyl β‐D‐thiogalactopyranoside (IPTG), followed by incubation at 18°C and 200 rpm for 18 h. Cells were harvested by centrifugation (10 min, 4 C, 6000 × g), and the resulting pellets were stored at −20°C until further purification.

### 4.2 Purification of Peptidases NosP/MicP2

All steps for protein purification were performed at 4 °C or on ice. Cells containing the expressed His_6_‐peptidase were thawed, resuspended in a lysis buffer (100 mM Tris, 150 mM NaCl, 10% glycerol (w/v), pH 7.5) and lysed by sonication (Sonopuls HD ultrasonic homogenizer, 10 min, 70% amplitude, pulse 3 s on/off). To the lysed cells, imidazole was added for a final concentration of 10 mM, and the cells were centrifuged (20 min, 4°C, 10 000 × g). The supernatant was incubated with 1 mL PureCube Ni‐NTA agarose at 4°C for 1 h. Subsequently, the resin was washed twice with 5 mL lysis buffer containing 20 mM imidazole and once with 5 mL lysis buffer containing 50 mM imidazole. The peptidase was eluted twice with 1 mL lysis buffer containing 250 mM imidazole. The enzyme was concentrated using Amicon Ultra‐4 centrifugal filters and used immediately.

### 4.3 Purification of Linear MvdE

Cells containing the expressed His_6_‐MBP‐MvdE fusion protein were thawed, resuspended in a lysis buffer (100 mM Tris, 150 mM NaCl, 10% glycerol (w/v), pH 7.5), and sonicated at 70% amplitude for 10 min using 3 s pulse on and 3 s pulse off. To the lysed cells, imidazole was added for a final concentration of 10 mM, and the cells were centrifuged (30 min, 4°C, 10 000 × g). The supernatant was transferred to a new tube, DNase (1U/mL supernatant) was added and incubated for 1 h at 4°C. Further purification was done using an ÄKTA pure system (FPLC) with buffer A (lysis buffer, 100 mM Tris, 150 mM NaCl, 10% glycerol (w/v), pH 7.5) and buffer B (elution buffer, 100 mM Tris, 150 mM NaCl, 10% glycerol (w/v), 500 mM imidazole, pH 7.5) and a flow rate of 1 mL/min. The sample was applied to a HisTrap HP 1 mL column from Cytiva that was preequilibrated with 2% buffer B. After sample application, the column was washed first with 10 column volumes (CV) of 2% buffer B, followed by 15 CV of 4% buffer B. The protein was eluted with 50% of buffer B over 10 CV, followed by a final wash step at 100% buffer B for 10 CV. The elution fractions were analyzed by SDS‐PAGE, fractions containing the protein were concentrated using Amicon Ultra‐4 centrifugal filters. To cleave His_6_‐MBP from the fusion protein, 70 µg of fusion protein was incubated with 500 U TEV protease (roboklon) overnight at 4°C. The reaction mix was again applied to the HisTrap column using the same program. The flow‐through was collected, and fractions were analyzed by SDS‐PAGE. Fractions containing the cleaved microviridin precursor peptide were concentrated using Amicon Ultra‐4 centrifugal filters.

### 4.4 In vitro Cyclization of MvdE

Enzyme production and purification, as well as microviridin cyclization assays and was carried out as previously reported with the exception that 150 μg precursor peptide and 37.5 μg of each ATP‐grasp ligase were used [28].

### 4.5 In vitro Peptidase Assays

For the endopeptidase assay, the cyclic precursor peptide (40 µM) was incubated with NosP (80 µM) in 50 mM Tris, 200 mM NaCl, 10% (w/v) glycerol at pH 8.0 and 37°C. A no‐enzyme control was included. The assay was performed three independent times. For the aminopeptidase assay, a mixture of microviridins N7‐N9 (25 µM), purified from *N. punctiforme* PCC 73 102, was incubated with NosP or MicP2 (5 µM) in the same buffer at pH of 7.7 and 37°C. The assays without the addition of a peptidase (no‐enzyme control) after 2 h for NosP and 15 min for MicP2 served as control. The reaction was quenched by the addition of acetonitrile (1:1).

### 4.6 Mass Spectrometric Analysis of Microviridins

For MALDI‐TOF MS, HPLC fractions were dried and resuspended in 2 μl acetonitrile and 8 μl 0.1% TFA. The samples were analyzed with the MicroflexLRF MALDI‐TOF MS system in positive ionization mode equipped with a nitrogen laser (*λ* = 337 nm). As a matrix solution, 3 mg/ml HCCA, dissolved in 84% acetonitrile, 13% ethanol, 3% ultrapure water, and 0.1% TFA was used. For mass spectrometry, 0.3 μl sample solution were spotted on the stainless steel MALDI target plate and covered with 0.3 μl HCCA matrix solution. With the samples dried, the target was inserted into the mass spectrometer. The laser power was set to 30%. Data was recorded with the FlexControl software (version 3.0; Bruker, Billerica, MA, USA) and analyzed with the mMass software (version 5.5.0).

Analytical HPLC‐HRMS was performed on a Thermo Scientific Vanquish Flex LC system coupled to an Orbitrap Exploris 240 mass spectrometer. The samples (5 µL injections) were separated on a Phenomenex Kinetex C18 (50 X 2.1 mm, 1.7 µm particle size, 100 Å pore size) heated to 40°C. HPLC separation was performed with the following standard methods (solvent A: H2O + 0.1% FA; solvent B: acetonitrile (ACN) + 0.1% FA, flow rate: 0.5 mL/min): 1 min at 5% B; 1–11 min from 5% to 100% B; 11–13 min at 100% B; 13–14 min from 100% to 5%. The mass spectrometer was run in positive ion mode with heated electrospray ionization (spray voltage: 3500 V, ion transfer tube temperature: 300°C, vaporizer temperature: 350°C, S‐lens RF level 70%). Full MS scans were acquired over an *m/z* range of 250–2000 at a resolution of 30,000 (AGC target: 2e5, maximum injection time: 54 ms). MS2 fragmentation was performed at a resolution of 15,000 (AGC target: 2e4, maximum injection time: 54 ms, isolation window: 1.0 *m/z*) with a normalized collision energy (NCE) of 30.

## Supporting Information

Additional supporting information can be found online in the Supporting Information section. **Supporting Figure S1**: Structures of all known class I microviridins for which a biosynthetic gene cluster can be identified. **Supporting Figure S2**: Sequence alignment of known and putative novel microviridinprecursor peptides. **Supporting Figure S3**: Sequence alignment of NosP with aminopeptidase N from *Escherichia coli* and the lanthipeptide leader‐processing peptidase AplP. **Supporting Figure S4**: Structure of the microviridin precursor peptide (MvdE) from *Nostoc punctiforme* PCC 73102 that was synthesized by SPPS. **S** **upporting Figure S5**: ESI‐mass spectrum of the chemically synthesized microviridin precursor peptide (MvdE). **Supporting Figure S6**: Purified proteins His6‐MBP‐MvdE, MvdE precursor peptide and His6‐NosP. **Supporting Figure S7**: Purified ATP‐grasp ligases used for in vitro assays. **Supporting Figure S8**: Chemoenzymatic synthesis of tricyclic MvdE precursor**. Supporting Figure S9**: LC‐MS spectrum of the heterologously produced precursor peptide GA‐MvdE**. Supporting Figure S10**: Incubation of the microviridin fraction with MicP2. **Supporting Table S1**: Vectors used in this study . **Supporting Table S2**: Strains used in this study. **Supporting Table S3**: Primers used in this study for Gibson Assembly. **Supporting Table S4**: LC‐MS results of NosP‐mediated cleavage of the microviridin precursor (GA‐MvdE) that were used for Figure 3d.

## Conflicts of Interest

The authors declare no conflicts of interest.

## Supporting information

Supplementary Material
