# Supplementary material for: A Bifunctional Protease Cleaves the Leader Peptide in the Biosynthesis of Class I Microviridins
Source: Chembiochem. 2026 Mar 31;27(7):e70297. doi: 10.1002/cbic.70297 (PMC13039773; doi:10.1002/cbic.70297)
Supplement: Supplementary file 1 — Supplementary Material [file CBIC-27-e70297-s001.pdf]

Supporting Information to:

# A Bifunctional Protease Cleaves the Leader Peptide in the Biosynthesis of Class I Microviridins

Nico Brüssow, Stella Scholz, Antje Stindt, Vincent Wiebach, Tino Damaszek, Roderich D. Süssmuth, Elke Dittmann, Martin Baunach

## Supplementary materials and methods

## Supplementary Tables:

**Table S1**. Vectors used in this study

**Table S2**. Strains used in this study

**Table S3**. Primers used in this study for Gibson Assembly

**Table S4**. LC-MS results of NosP-mediated cleavage of the microviridin precursor (GA-MvdE) that were used for Figure 3d

## Supplemental Figures:

**Figure S1**. Structures of all known class I microviridins for which a biosynthetic gene cluster can be identified.

**Figure S2**. Sequence alignment of known and putative novel microviridin precursor peptides.

**Figure S3**. Sequence alignment of NosP with aminopeptidase N from *Escherichia coli* and the lanthipeptide leader-processing peptidase AplP.

**Figure S4**. Structure of the microviridin precursor peptide (MvdE) from *Nostoc punctiforme* PCC 73102 that was synthesized by SPPS.

**Figure S5**. ESI-mass spectrum of the chemically synthesized microviridin precursor peptide (MvdE).

**Figure S6**. Purified proteins $His_6$-MBP-MvdE, MvdE precursor peptide and $His_6$-NosP.

**Figure S7**. Purified ATP-grasp ligases used for *in vitro* assays.

**Figure S8**. Chemoenzymatic synthesis of tricyclic MvdE precursor.

**Figure S9**. LC-MS spectrum of the heterologously produced precursor peptide GA-MvdE.

**Figure S10**. Incubation of the microviridin fraction with MicP2.

## Supplementary materials and methods

**Reagents, Solvents, and Chromatographic Conditions.** Commercially available reagents (Carl Roth GmbH and Co. KG, Karlsruhe, Germany; Sigma-Aldrich Taufkirchen, Germany; Iris Biotech GmbH, Marktredwitz, Germany; Orpegen, Heidelberg, Germany; ABCR, Karlsruhe, Germany; TCI, Eschborn, Germany; VWR International GmbH, Darmstadt, Germany; and Acros, Geel, Belgien) and solvents (Fisher Scientific-Acros, Schwerte, Germany) were used without further purification. If necessary, reactions were carried out under an atmosphere of nitrogen and dry solvents. Preparative HPLC was carried out on a 1260 Infinity (Agilent Technologies, Waldbronn, Germany) HPLC system with two reversed phase columns (Phenomenex Luna, 250 x 21.2 mm, particle size 10 µm and Phenomenex Gemini, 150 x 10.0 mm, particle size 5 µm). Analytical HPLC-HRMS spectra were recorded on a Orbitrap (Thermo Fisher Scientific, Waltham, Massachusetts, USA) hyphenated to an Agilent 1200 Series HPLC-System (Agilent Technologies, Waldbronn, Germany) equipped with a C18 column (50 x 2 mm, particle size 3 µm). HPLC-HRMS chromatograms were obtained with a solvent gradient of 0.1% formic acid in water (Solvent A) and 0.1% formic acid in acetonitrile (Solvent B). Following solvent gradient was followed: flow: 0.4 mL/min; 0-2 min 0 % MeCN, 2-20 min 0-100 % MeCN, 20-25 min 100% MeCN, 25-27 min 100-0 % MeCN.

**General Protocol for the Automated Solid-Phase Peptide Synthesis (SPPS).** Peptidyl resin 2-CTC resin (3 g, 0.98 mmol/g) was pre-swollen for 20 min in DMF in a manual solid phase peptide synthesis vessel (50 mL). After the solvent was drained, Fmoc-L-Arg(Pbf)-OH (3 mmol) and DIPEA (4.5 mmol) in DMF (40 mL) were added to the resin. The mixture was agitated for 2 h before the solvent was drained. The resin was rinsed with DMF (4 x 20 mL). Then a mixture of MeOH/DIPEA/DMF (1:1:8, 50 mL) was added to cap the remaining 2-chlorotrityl chloride on the resin. The mixture was agitated under nitrogen for 0.5 h. Then the solvent was drained, and the resin was washed with DMF (4 x 25 mL) before drying under vacuum. The resin loading was determined to be 0.18 mmol/g. The automated SPPS was carried out on a CEM synthesizer (Liberty Prime, CEM, Kamp-Lintfort, Germany). The temperature of the reactions during synthesis was controlled via microwave irradiation and set to 50 °C. Amino acid building blocks where prepared in a 0.5 M solution in DMF. DIC (2 M in DMF) and Oxyma Pure (0.25 M in DMF) where used for coupling reactions. For capping, a capping solution of $Ac_2O$ and pyridine (2:3, v/v) in DMA was used. The Fmoc group was removed according to **Method A**. The following side chain protected amino acid building block was coupled to the deprotected resin according to **Method B**, or **Method C**. Capping followed the procedure of **Method D**.

**Method A** (Removal of the Fmoc group): A solution of 20 % (v/v) piperidine in DMF (24 mL) was added to the resin and the resulting suspension was heated for 5 min. The solution was drained, and the resin was washed with DMF (3 x 20 mL). **Method B** (Amino acid single coupling): Amino acid building block (8 mL), DIC (8 mL) and Oxyma Pure (8 mL) were added to the resin and heated for 30 min under Nitrogen agitation. Then, the reaction solution was drained, and the resin was rinsed with DMF (3 x 24 mL). **Method C** (Amino acid double coupling): Amino acid building block (8 mL), DIC (8 mL) and Oxyma Pure (8 mL) were added to the resin and heated for 30 min under Nitrogen agitation. The reaction solution was drained and replaced with amino acid building block (8 mL), DIC (8 mL) and Oxyma Pure (8 mL) and heated for 30 min. Then the reaction solution was drained, and the resin was rinsed with DMF (3 x 24 mL). **Method D** (capping): To the resin 20 mL capping solution were added and heated for 10 min under nitrogen agitation. Then the capping solution was drained, and the resin was rinsed with DMF (3 x 24 mL). Following the automated synthesis, the resulting peptidyl resin was dried under vacuum. The dry resin was equally divided into four empty syringes. To each syringe, 5 mL cleavage solution (TFA/TIPS/DODT/water, 92.5/2.5/2.5/2.5) were added and

shaken for 2 h at room temperature. The resulting combined supernatant was added to 50 mL ice cold $Et_2O$ and cooled for 30 min at 4ºC. The resulting mixture was centrifuged for 15 min at 20000 rpm before removing the supernatant from the pellet. The precipitated peptide was redissolved in water and freeze-dried prior to purification. All crude peptide samples were dissolved in a mixture of water/MeCN (1:1) before HPLC purification steps.

**Synthesis of microviridin precursor peptide MvdE.** Peptidyl resin 2-CTC resin (3 g, 0.98 mmol/g) was pre-swollen for 20 min in DMF in a manual solid phase peptide synthesis vessel (50 mL). After the solvent was drained, Fmoc-L-Arg(Pbf)-OH (3 mmol) and DIPEA (4.5 mmol) in DMF (40 mL) were added to the resin. The mixture was agitated for 2 h and before the solvent was drained. The resin was rinsed with DMF (4 x 20 mL). Then a mixture of MeOH/DIPEA/DMF (1:1:8, 50 mL) was added to cap the remaining 2-chlorotrityl chloride on the resin. The mixture was agitated under nitrogen for 0.5 h. Then the solvent was drained, and the resin was washed with DMF (4 x 25 mL) and dried under vacuum. The resin loading was determined to be 0.18 mmol/g. The Fmoc group was removed according to **Method A**. Fmoc-Asp(Boc)-OH (4 eq.) was coupled to the deprotected resin according to **Method B**. The coupling procedures were repeated until the following sequence was obtained (from C- to N-terminus): CTC-Arg(Pbf)-Asp(tBu)-Glu(tBu)-Leu-Asp(tBu)-Ser(tBu)-Pro-Phe-Lys(Boc)-Tyr(tBu)-Thr(tBu)-Trp(Boc)-Pro-Val-Glu(tBu)-Thr(tBu)-Gly-Glu(tBu)-Thr(tBu)-Ala-Gln(Trt)-Glu(tBu)-Glu(tBu)-Leu-Phe-Arg(Pbf)-Ala-Phe-Phe-Pro-Val-Ala-$NH_2$. Following coupling steps were performed according to **Method C** until this sequence was obtained: CTC-Arg(PbF)-Asp(tBu)-Glu(tBu)-Leu-Asp(tBu)-Ser(tBu)-Pro-Phe-Lys(Boc)-Tyr(tBu)-Thr(tBu)-Trp(Boc)-Pro-Val-Glu(tBu)-Thr(tBu-Gly-Glu(tBu)-Thr(tBu)-Ala-Gln(Trt)-Glu(tBu)-Glu(tBu)-Leu-Phe-Arg(Pbf)-Ala-Phe-Phe-Pro-Val-Ala-Val-Val-Asp(tBu)-Val-Thr(tBu)-Lys(Boc)-Val-Thr(tBu)-Asn(Trt)-Thr(tBu)-Asn(Trt)-Thr(tBu)-Pro-Met-$NH_2$. Cleavage of the peptide was carried out in a solution of TFA/DODT/TIPS/water (92.5/2.5/2.5/2.5, v/v) and shaken for 2 h at room temperature. The resulting combined supernatant was added to 50 mL ice cold $Et_2O$ and cooled for 30 min at 4ºC. The resulting mixture was centrifuged for 15 min at 20000 rpm before removing the supernatant from the pellet. The freeze-dried pellet was purified with reverse-phase HPLC (flow: 40 mL/min; 0-30 min 5-60 %MeCN 30-34 min 60-100 % MeCN, **Rt** = 20.5-21.8 min). The resulting HPLC fractions were freeze-dried to obtain a colorless solid. The obtained solid was redissolved in water and purified with reverse-phase HPLC (flow: 10 mL/min; 0-60 min 5-50 %MeCN, **Rt** = 38.5-39.5 min. The final peptide was obtained as a with solid (7.0 mg).

***In vitro* MicP2 assay with extended incubation time (0-72 h).** For the aminopeptidase assay, a mixture of microviridins N7-N9 (500 µM), purified from *N. punctiforme* PCC 73102, was incubated with MicP2 (5 µM) in assay buffer (50 mM Tris, 200 mM NaCl, 1 mM DTT, 10% (w/v) glycerol, pH 8.5) at 30 °C for 0-72 h. The reaction was quenched by the addition of acetonitrile (1:1). Measurements of quenched MicP2 reactions with the MVD mixture were conducted on an LTQ-Orbitrap XL mass spectrometer (Thermo Fisher Scientific, Bremen, Germany) coupled to an analytical Agilent 1200 HPLC system (Agilent, Waldbronn, Germany) equipped with a Grom-Sil-120-ODS-4-HE column (50.0 × 2.0 mm; Grace, Deerfield, IL, USA). The HPLC-gradient was set to increase linearly from 20–100 % solvent B in 10 min, hold at 100 % B for 3 min and following isocratic at 20 % B for 4 min. Measurements were conducted in positive ionization mode in a scan-range from 200-2000 m/z. All measurements were conducted in FTMS mode with a resolution of R = 60,000. Collected data of MS experiments was analyzed using the Thermo Xcalibur 2.2. software.

## Supplementary Tables

**Table S1**. Vectors used in this study.

| Name | Source | Resistance marker |
|---|---|---|
| pET28a | Sigma-Aldrich, St. Louis, MO, USA | Kanamycin |
| pETMBP_1a | EMBL-made vector by Gunter Stier (provided by lab of Prof. Dr. Süßmuth, TU Berlin, Germany) | Kanamycin |

**Table S2**. Strains used in this study.

| Organism | Phylum | Source |
|---|---|---|
| *E. coli* XL1-blue | proteobacteria | Agilent Technologies, Waldbronn, Germany |
| *E. coli* LOBSTR | proteobacteria | Kerafast, Inc., Boston, MA, USA |
| *Nostoc punctiforme* PCC 73102 | cyanobacteria | Pasteur Culture Collection |

**Table S3**. Primers used in this study for Gibson Assembly.

| Name | Sequence (5' to 3') | Description |
|---|---|---|
| S064_MvdPrecursor_F | TGAGAATCTTTATTTTCAGGGCGCCatgcctacaaacacagtcaaaac | MvdE (NPUN_RS38845) |
| S065_MvdPrecursor_R | TCGACGGAGCTCGAATTCGGATCCGttatctatcttccaaatctgaaggaaact | MvdE (NPUN_RS38845) |
| S005 _Npun_F2817_AP_F | TGGTGCCGCGCGGCAGCCACatgtcgaagtcttattttgatacagataataac | NosP (NPUN_RS14335) |
| S006_ Npun_F2817_AP_R | TCCACCAGTCATGCTAGCCActatttagatttggcctccaagttttc | NosP (NPUN_RS14335) |

**Table S4**. LC-MS results of NosP-mediated cleavage of the microviridin precursor (GA-MvdE) that were used for Figure 3d.

| Peptide | Ion | Calculated mass (m/z) | Observed mass (m/z) | Intensity (0.5 h) | Intensity (1 h) | Intensity (20 h) |
|---|---|---|---|---|---|---|
| N0 | $[M+1H]^{1+}$ | 1316.628 | - | - | - | - |
| N1 | $[M+1H]^{1+}$ | 1502.708 | - | - | - | - |
| N2 | $[M+1H]^{1+}$ | 1599.758 | - | - | - | - |
| N3 | $[M+2H]^{2+}$ | 849.918 | 849.919 | 5.63E+04 | 1.08E+05 | 8.06E+05 |
| N4 | $[M+2H]^{2+}$ | 914.438 | 914.438 | - | - | 1.07E+06 |
| N5 | $[M+2H]^{2+}$ | 964.963 | 964.966 | 1.06E+05 | 1.58E+05 | 8.24E+05 |
| N6 | $[M+2H]^{2+}$ | 993.473 | 993.473 | - | - | 4.53E+05 |
| N7 | $[M+2H]^{2+}$ | 1057.993 | 1057.997 | - | - | 1.22E+06 |
| N8 | $[M+2H]^{2+}$ | 1108.518 | 1108.519 | 2.15E+05 | 5.32E+05 | 2.63E+07 |
| N9 | $[M+2H]^{2+}$ | 1144.038 | 1144.038 | 2.40E+06 | 5.10E+06 | 5.39E+06 |
| N10 | $[M+2H]^{2+}$ | 1208.067 | 1208.065 | 3.41E+05 | 5.91E+05 | 6.28E+05 |
| N11 | $[M+2H]^{2+}$ | 1272.587 | 1272.586 | 2.21E+05 | 5.29E+05 | 1.26E+06 |
| N12 | $[M+2H]^{2+}$ | 1337.107 | 1337.108 | 1.38E+05 | 3.23E+05 | 4.32E+06 |
| N13 | $[M+2H]^{2+}$ | 1393.652 | 1393.656 | 8.04E+05 | 1.46E+06 | 7.24E+06 |
| N14 | $[M+2H]^{2+}$ | 1467.187 | 1467.188 | 1.55E+05 | 4.81E+05 | 1.80E+07 |
| N15 | $[M+3H]^{3+}$ | 1030.494 | 1030.495 | 3.34E+05 | 8.42E+05 | 1.07E+06 |
| N16 | $[M+3H]^{3+}$ | 1054.174 | 1054.174 | 1.44E+06 | 3.04E+06 | 8.15E+05 |
| N17 | $[M+3H]^{3+}$ | 1103.194 | 1103.192 | 1.08E+05 | 1.63E+05 | - |
| N18 | $[M+3H]^{3+}$ | 1152.217 | - | - | - | - |
| N19 | $[M+3H]^{3+}$ | 1184.567 | - | - | - | - |
| N20 | $[M+3H]^{3+}$ | 1217.591 | 1217.595 | 1.68E+05 | 7.19E+05 | 1.09E+07 |
| N21 | $[M+3H]^{3+}$ | 1241.271 | 1241.277 | 1.97E+06 | 4.41E+06 | 8.73E+05 |
| N22 | $[M+3H]^{3+}$ | 1274.294 | 1274.280 | 2.52E+05 | - | - |
| N23 | $[M+3H]^{3+}$ | 1307.317 | - | - | - | - |
| N24 | $[M+3H]^{3+}$ | 1345.657 | 1345.661 | - | - | 1.66E+06 |
| N25 | $[M+3H]^{3+}$ | 1378.681 | 1378.679 | 1.37E+06 | 2.19E+06 | - |
| N26 | $[M+3H]^{3+}$ | 1412.364 | 1412.362 | 6.48E+05 | 9.45E+05 | 1.28E+06 |
| N27 | $[M+4H]^{4+}$ | 1091.550 | 1091.546 | - | - | 3.03E+05 |
| N28 | $[M+4H]^{4+}$ | 1116.315 | - | - | - | - |
| N29 | $[M+4H]^{4+}$ | 1141.577 | - | - | - | - |
| N30 | $[M+4H]^{4+}$ | 1170.087 | - | - | - | - |
| N31 | $[M+4H]^{4+}$ | 1195.350 | - | - | - | - |
| N32 | $[M+4H]^{4+}$ | 1219.612 | - | - | - | - |
| N33 | $[M+4H]^{4+}$ | 1252.375 | 1252.379 | - | - | 8.06E+06 |
| N34 | $[M+4H]^{4+}$ | 1270.132 | 1270.132 | - | 3.07E+06 | 1.16E+06 |
| full-length | $[M+4H]^{4+}$ | 1284.387 | 1284.392 | 1.09E+08 | 1.12E+08 | 3.30E+06 |

## Supplemental Figures

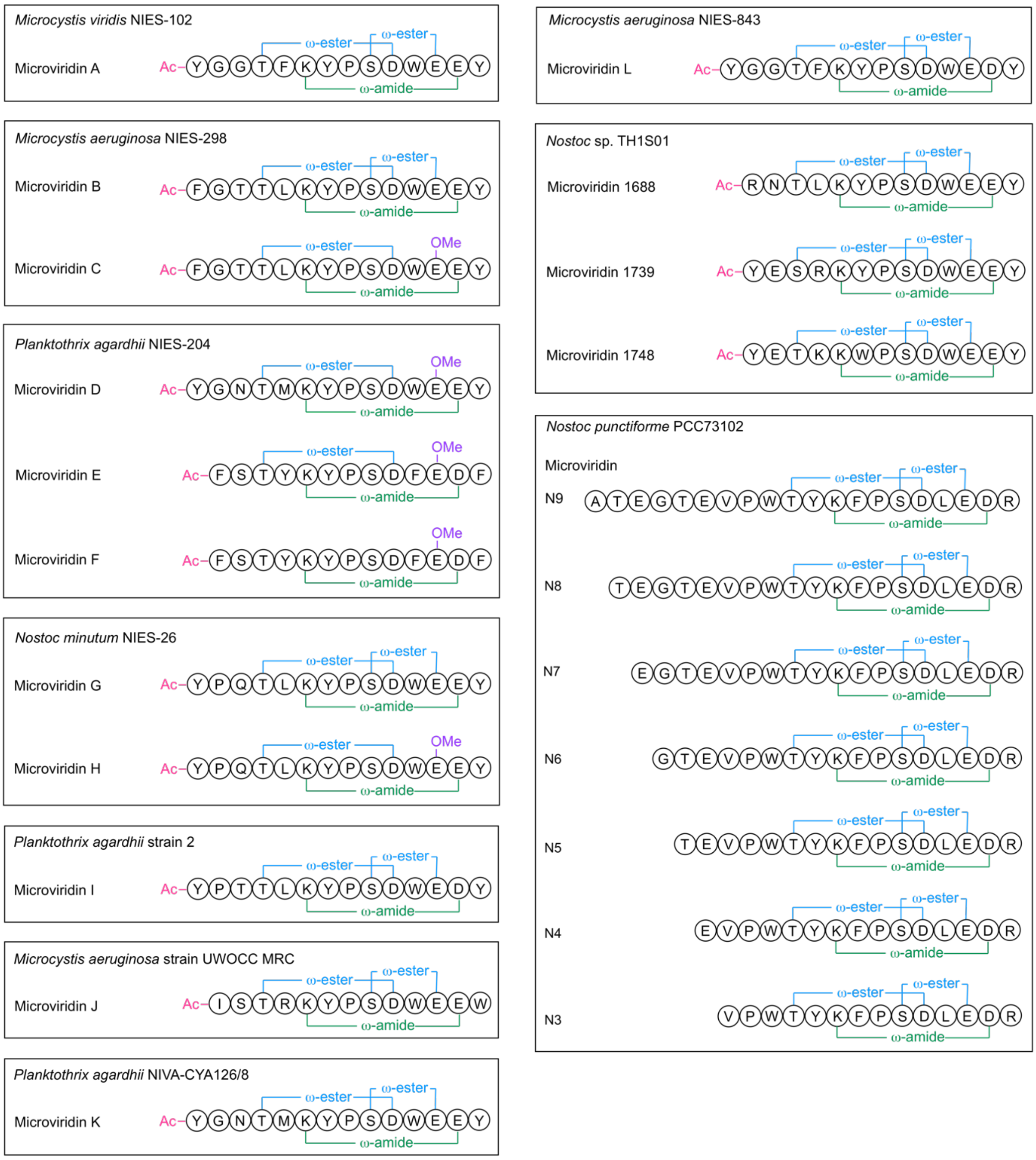


**Figure S1**. Structures of all known class I microviridins for which a biosynthetic gene cluster can be identified. The corresponding leader peptide sequences were used for the alignment that is depicted in Figure 1a.

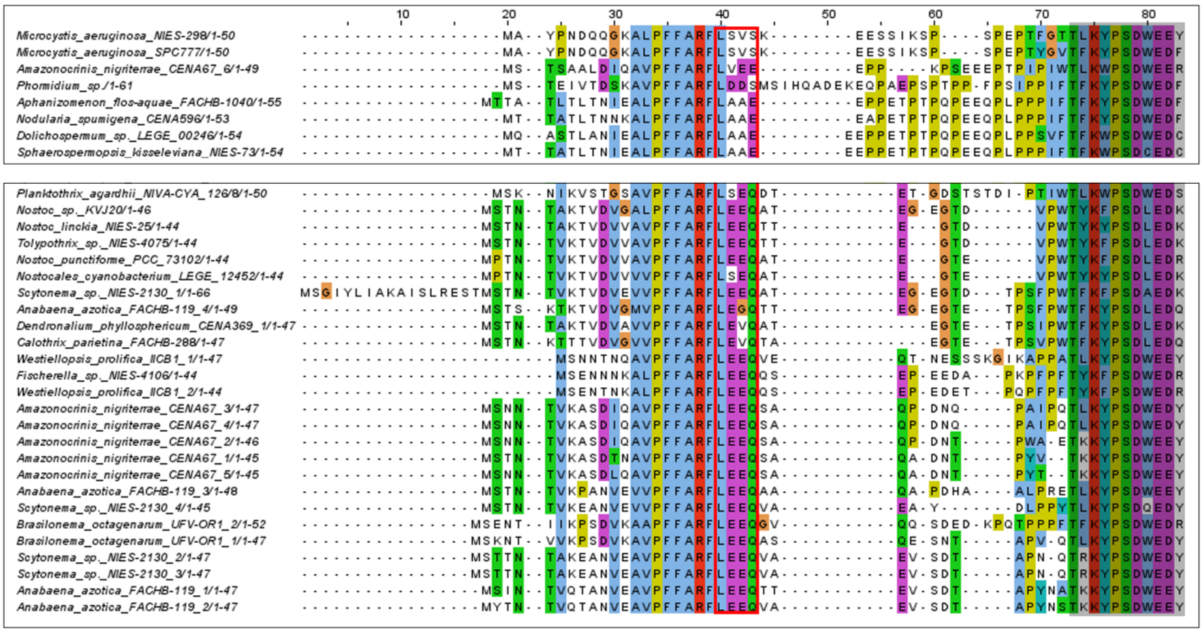


**Figure S2.** Sequence alignment of known and putative novel type I microviridin precursor peptides further hints to the existence of two distinct groups of leader peptides. The first group is characterized by a proline-rich sequence at the C-terminus of the leader peptide, whereas the second group is clearly distinct and showed congruence only in the conserved PFFARFL motif, which is essential for the activation of the ATP grasp ligases MvdC and MvdD. In addition to that, the proposed endopeptidase recognition motif LEEQ (marked in red) is much more conserved in the second group.

```
ePepN     AVDFFNMGAMENKGLNIFNSKYVLARTDTATDKDYLDIERVIGHEYFHNWTGNRVTCRDWFQLSLKEGLTVFRDQ
NosP      -VDDFIFGGMENTSTTLLTDRCLLD---ERAALDNRNTESLVVHELAHQWFGDLVVIKHWSHAWIKEGMASYSEV
AplP      LVAELPAAAMENAACVTLSESLARP--GTADPAARLRRSGVLLHELAHMWFGDLVTMRWWDDLWLNESFAVWAAV
           *   :   ..***  .    :..                  . ::  **   *  * *:  *. :  *  .    ::*.:: :
```

**Figure S3**. Sequence alignment of NosP with the characterized aminopeptidase N from *Escherichia coli* and the lanthipeptide leader-processing peptidase AplP. Putative catalytic motifs are marked in red. The conserved GAMEN motif, which contains a highly conserved Glu residue that is suggested to contribute to the exopeptidase activity of various proteases through interaction of its terminal carboxylic group with the N-terminus of the substrate is highlighted in yellow. The putative Zn binding residues of the highly conserved catalytic zinc-binding motif (HEXXH-$X_{18}$-E) are highlighted in green. GenBank accession numbers are listed as follows: ePepN, AAA24317.1; NosP, WP_012409343.1; AplP, AHB63590.1.

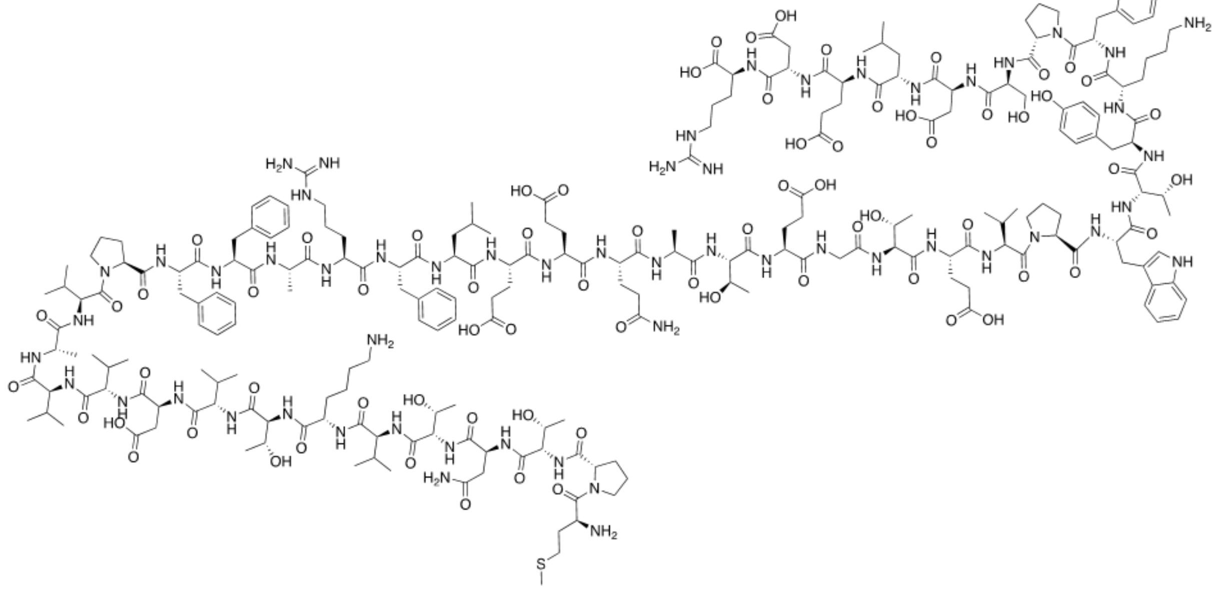

**Figure S4.** Structure of the microviridin precursor peptide (MvdE) from *N. punctiforme* PCC 73102 that was synthesized by SPPS.

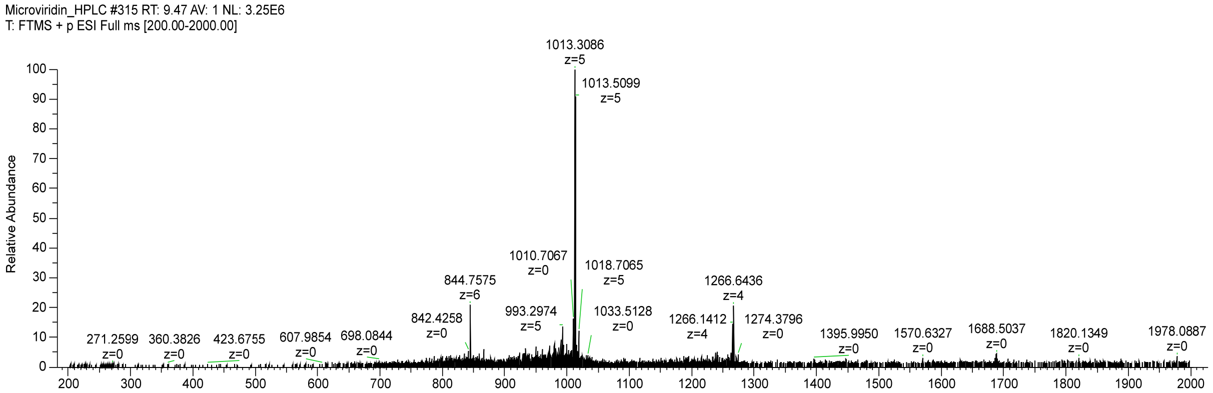


**Figure S5.** ESI-mass spectrum of the chemically synthesized microviridin precursor peptide (MvdE; $C_{231}H_{352}N_{55}O_{71}S$). Calculated m/z ($[M+5H]^{5+}$) 1013.3077, measured m/z: 1013.3086.

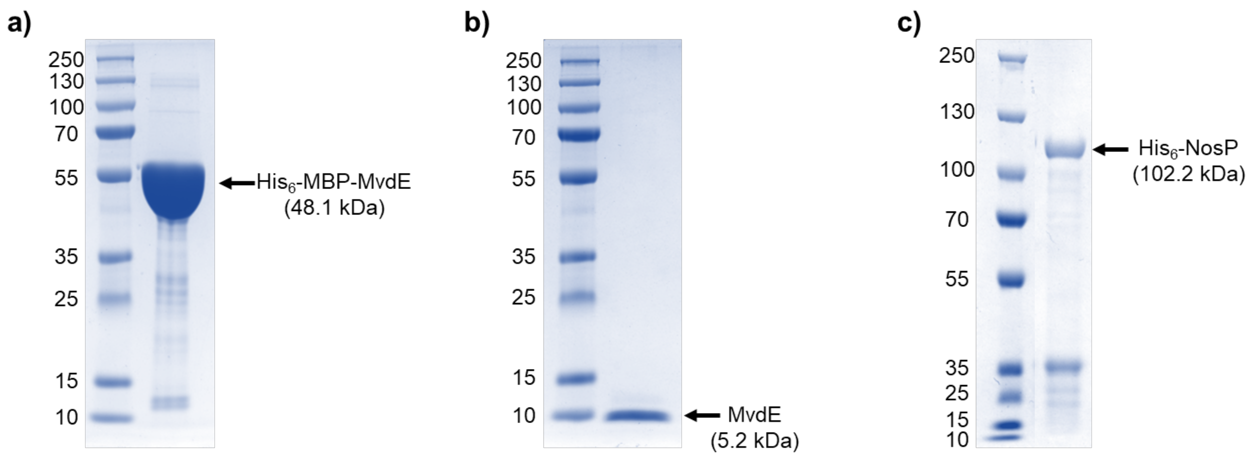


**Figure S6**. Purified proteins. a) Purified His$_6$-MBP-MvdE. b) Purified MvdE precursor peptide after MBP removal by a TEV protease. c) Purified His$_6$-NosP used for *in vitro* assays.

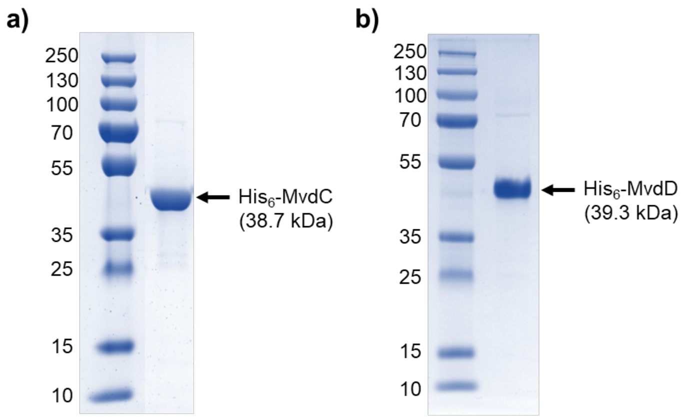


**Figure S7**. Purified ATP-grasp ligases used for *in vitro* assays. a) Purified His$_6$-MvdC. b) Purified His$_6$-MvdD.

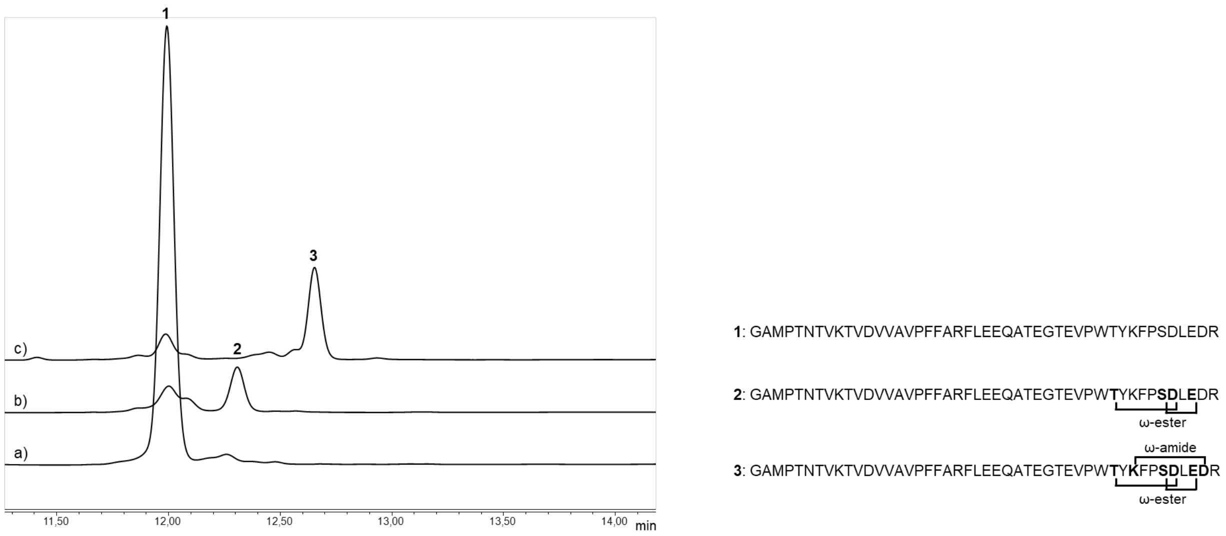


**Figure S8**. Chemoenzymatic synthesis of tricyclic MvdE precursor. a) Heterologously expressed precursor peptide GA-MvdE (**1**, control). b) Addition of the enzyme MvdD produces the bicyclic precursor **2**. c) Addition of both enzymes MvdC and MvdD produces the tricyclic precursor **3**.

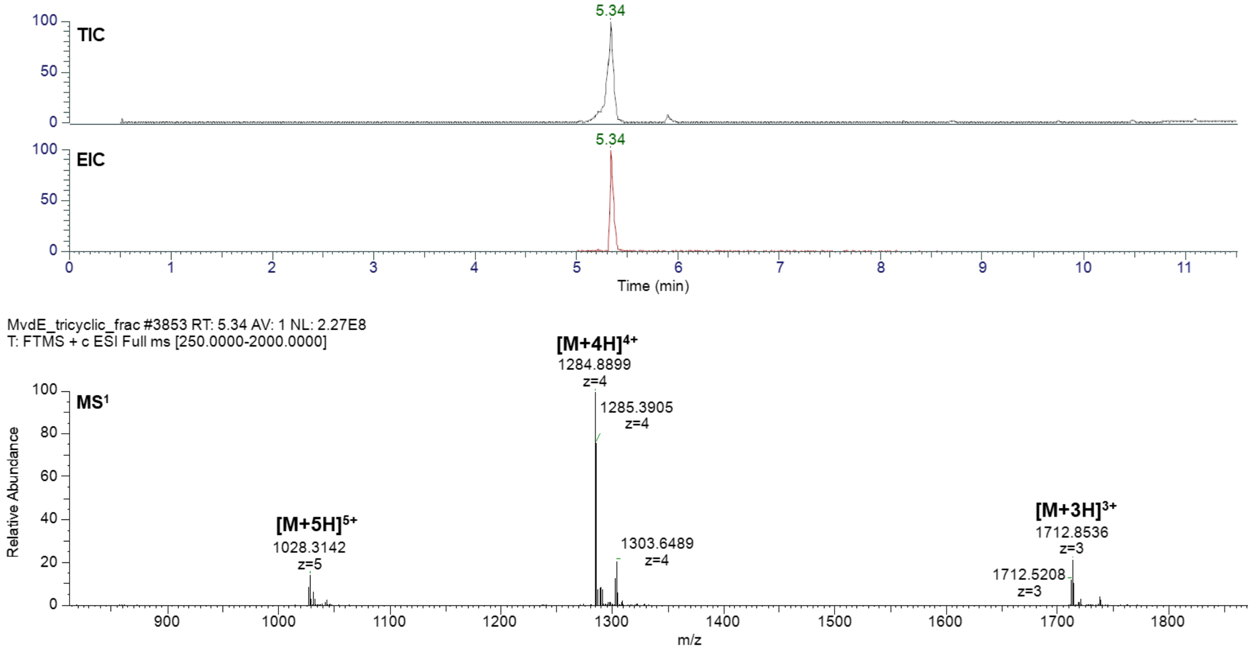


**Figure S9**. ESI-mass spectrum of the heterologously produced precursor peptide GA-MvdE. The cyclic precursor peptide showed at a retention time of 5.34 min a major peak in the MS spectrum at m/z 1284.890 corresponding to the expected $[M+4H]^{4+}$ species.

a)

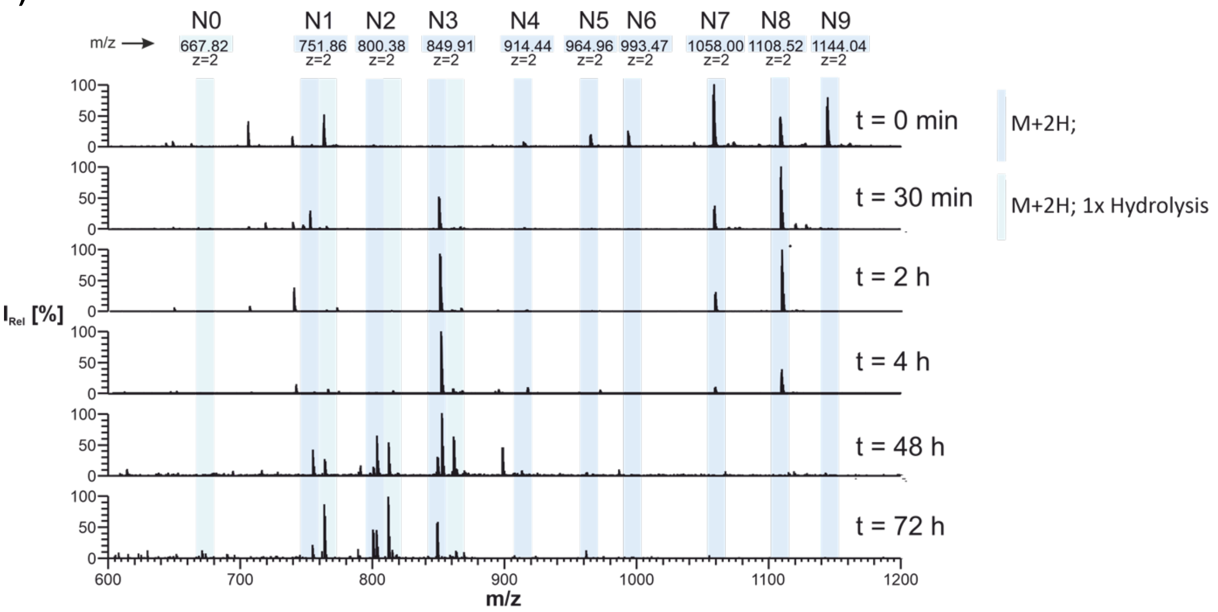


b)

| $[M+2H]^{2+}$ | Measured | Calculated | Δ ppm |
|---|---|---|---|
| N3 (hydrolysis) | 858.92104 | 858.9227 | -1.93 |
| N3 | 849.91585 | 849.9174 | -1.82 |
| N2 (hydrolysis) | 809.38715 | 809.3884 | -1.54 |
| N2 | 800.38168 | 800.3832 | -1.90 |
| N1 (hydrolysis) | 760.86083 | 760.8621 | -1.67 |
| N1 | 751.85550 | 751.8568 | -1.73 |
| N0 (hydrolysis) | 667.82076 | 667.8224 | -2.46 |
| N0 | n. o. | 658.8171 | n. a. |

**Figure S10.** Incubation of the microviridin fraction with MicP2. a) Incubation of the microviridin fraction with MicP2 results in the formation of microviridin species with shortened N-termini (N2-N1) and even the removal of the complete N-terminal overhang (N0) upon extended incubation times. With extended incubation times spontaneous hydrolysis of microviridins' ester bonds leads to an increase in hydrolyzed microviridin species, a phenomenon which is known for this compound class. b) Measured and calculated m/z for microviridin species ($[M+2H]^{2+}$) with shortened N-termini (N3-N0) intact and with one hydrolyzed ester bond. n. o. – not observed; n. a. – not applicable.
